# Effects of Wearable Devices on Parkinson Disease: Systematic Review and Meta-Analysis of Randomized Controlled Trials Within the International Classification of Functioning, Disability, and Health Framework

**DOI:** 10.2196/85914

**Published:** 2026-03-18

**Authors:** Jiarong Wu, Wanli Zang, Mingqing Fang, Ningkun Xiao, Xianzuo Zhang, Su Wang, Qiuxia Zhang

**Affiliations:** 1 School of Physical Education Soochow University Suzhou, Jiangsu China; 2 Xiangya School of Medicine Central South University Changsha, Hunan China; 3 Laboratory for Brain and Neurocognitive Development, Department of Psychology Institution of Humanities Ural Federal University Yekaterinburg Russian Federation; 4 Department of Orthopedics The First Affiliated Hospital of USTC, Division of Life Sciences and Medicine University of Science and Technology of China Hefei, Anhui China; 5 College of Sports Science and Health University of Harbin Sport Harbin, Heilongjiang China

**Keywords:** gait performance, balance function, wearable devices, meta-analysis, International Classification of Functioning, Disability and Health (ICF)

## Abstract

**Background:**

Parkinson disease (PD) impairs gait, balance, and quality of life, and wearable devices have been proposed to support rehabilitation, but evidence for their clinical efficacy remains uncertain.

**Objective:**

This study aimed to evaluate, within the International Classification of Functioning, Disability, and Health (ICF) framework, the effects of wearable-device interventions on gait performance, balance, and health-related quality of life in people with PD by conducting a systematic review and meta-analysis of randomized controlled trials (RCTs).

**Methods:**

We searched PubMed, Web of Science, Cochrane Library, Embase, and ClinicalTrials.gov from inception to November 18, 2025, for RCTs in people with PD comparing wearable-device interventions with control conditions. We used Hartung-Knapp random-effects models to pool mean differences (MDs) or standardized mean differences (SMDs) and reported 95% prediction intervals when ≥3 studies were pooled. Risk of bias was assessed using the Cochrane Risk of Bias (RoB) tool, and certainty of evidence was rated using Grading of Recommendations Assessment, Development, and Evaluation (GRADE).

**Results:**

Nine RCTs involving 260 participants were included. Wearable devices produced a small improvement in stride length (MD 0.10 meter, 95% CI 0.03-0.17), but there was no clear benefit for the 10-Meter Walk Test time (MD 0.04 second, 95% CI –0.06 to 0.15). Double support time showed no reduction (MD –1.59% gait cycle, 95% CI –3.79 to 0.61). Freezing of gait (Freezing of Gait Questionnaire [FOG-Q] and New Freezing of Gait Questionnaire [NFOG-Q]) did not significantly improve (SMD –0.24, 95% CI –0.72 to 0.24). Motor severity (Unified Parkinson Disease Rating Scale Part III [UPDRS III]) showed a small, nonsignificant trend favoring wearable devices (MD –2.16 points, 95% CI –4.39 to 0.07). For balance, pooled results from the Berg Balance Scale (BBS), Mini Balance Evaluation Systems Test (Mini-BESTest), and Performance-Oriented Mobility Assessment Balance Subscale (POMA balance) suggested a borderline effect (SMD 0.48, 95% CI –0.02 to 0.98). Wearable devices did not meaningfully improve Parkinson Disease Questionnaire (PDQ) scores (SMD –0.28, 95% CI –0.74 to 0.17), EQ-5D utility (MD 0.10, 95% CI –0.24 to 0.44), or Falls Efficacy Scale–International (FES-I) scores (MD –0.04, 95% CI –1.10 to 1.02). Prediction intervals frequently crossed the null, suggesting effects may vary by setting and population.

**Conclusions:**

Wearable device interventions for Parkinson disease produced a small improvement on average in stride length, with no consistent benefits for other gait outcomes, balance, or patient-centered outcomes. By integrating ICF mapping with Hartung-Knapp meta-analysis, prediction intervals, and GRADE, and avoiding pooling of conceptually distinct gait measures used in prior reviews, this review clarifies where evidence is most consistent, supports using wearables as adjuncts to rehabilitation, and underscores the need for larger, longer RCTs with standardized outcomes to determine who benefits and how to implement them.

**Trial Registration:**

PROSPERO CRD42024585686; https://www.crd.york.ac.uk/PROSPERO/view/CRD42024585686

## Introduction

Parkinson disease (PD) is the second most common neurodegenerative disorder, characterized by the loss of dopaminergic neurons in the substantia nigra and the presence of Lewy bodies containing α-synuclein [1]. Globally, the estimated number of patients with PD has increased from over 6 million in 1990 to more than 6 million in 2016 [2], and it is expected to double, reaching over 12 million by 2040 [3]. PD poses significant health challenges to patients and places considerable burdens on health care systems and the economy, primarily due to productivity loss and rising health care costs. As the disease progresses, motor symptoms such as bradykinesia, muscle rigidity, resting tremor, and postural instability become more pronounced [4-7]. These changes lead to secondary issues, including diminished walking ability (eg, reduced speed, stride length, and step frequency), increased fall risk, and severe limitations in community participation, which significantly impair patients’ quality of life [8-10]. Therefore, restoring walking and balance functions is a crucial aspect of PD rehabilitation, contributing to improved quality of life and independence for patients. While many studies have shown that levodopa can increase stride length and walking speed, treatment options become limited as the disease progresses. Often, after 3-5 years of levodopa use, the duration of its effect decreases, resulting in a worsening of symptoms before the next dose [11,12]. Moreover, long-term use of dopaminergic medications may lead to reduced efficacy and adverse effects such as dyskinesia [13], underscoring the need for alternative and promising PD treatments, such as wearable devices [14-16].

Wearable cueing and sensor-based devices provide a lightweight and portable means of delivering external cues or monitoring motor symptoms in everyday environments, without requiring the continuous presence of health care professionals [17-20]. In PD, these technologies have been used to provide auditory, visual, or somatosensory cues during gait, to quantify tremor and bradykinesia, and to track gait abnormalities, balance, activity levels, and sleep patterns in real time [21-23]. Among these technologies, inertial measurement units (IMUs)—typically combining triaxial accelerometers and gyroscopes into body-worn sensors—have become the most widely used platform for quantitative mobility assessment in both in-clinic and home-based settings [24]. Recent clinical and experimental studies suggest that such devices can acutely improve selected gait parameters and support more individualized adjustment of pharmacological or stimulation therapies [25]. The growing number of clinical and research applications has also prompted international initiatives, such as Movement Disorder Society task forces and national guidance (eg, the 2023 National Institute for Health and Care Excellence recommendations on device-based monitoring in PD), which highlight both the potential and the current uncertainties surrounding wearable technologies in routine care [26]. In addition, qualitative research indicates that long-term use is often limited by issues, such as physical discomfort, interface complexity, privacy concerns, and fluctuating motivation, which may reduce adherence and attenuate real-world effectiveness [27]. Overall, current evidence points to wearable devices as a promising but still incompletely understood adjunct in the management and rehabilitation of people with PD.

Despite this rapid technological development, the specific benefits of wearable devices in improving gait, balance, and quality of life for people with PD have not been fully confirmed. Many studies use observational or small pilot designs, involve heterogeneous device types and training regimens, and focus primarily on laboratory-based gait measures, with relatively less attention to balance, fear of falling, and broader patient-reported outcomes [28,29]. Existing reviews and meta-analyses have often combined wearable and nonwearable cueing interventions or have not applied contemporary random-effects methods, prediction intervals, or Grading of Recommendations Assessment, Development, and Evaluation (GRADE) to formally appraise the certainty of evidence [30]. Moreover, sensor-derived mobility metrics can be influenced by psychological, cognitive, environmental, and technical factors and may differ substantially between supervised laboratory assessments and unsupervised home recordings [30,31], further complicating interpretation of existing data [26,31]. As a result, the overall impact of wearable-device interventions on gait performance, balance, and health-related quality of life in PD remains uncertain.

Therefore, the aim of this study was to conduct a PRISMA (Preferred Reporting Items for Systematic Reviews and Meta-Analyses)-aligned systematic review and meta-analysis of randomized controlled trials (RCTs) to evaluate the effects of wearable-device interventions on gait performance, balance, and health-related quality of life in people with PD, interpreted within the International Classification of Functioning, Disability, and Health (ICF) framework. We specifically sought to (1) quantify the effects of wearable devices on key gait and motor outcomes (eg, stride length, short-distance gait speed, double support time (DST), freezing of gait, and Unified Parkinson Disease Rating Scale Part III (UPDRS III)), (2) examine their impact on balance and patient-reported outcomes such as quality of life and fear of falling, and (3) appraise the overall certainty of evidence using the GRADE approach. We hypothesized that wearable devices would yield modest improvements in specific gait parameters, whereas evidence for consistent benefits on balance and quality of life would be more limited.

## Methods

### Overview

This study is based on the ICF framework [32], analyzing the intervention effects of wearable devices on gait, balance, and quality of life in patients with PD. The Population, Intervention, Comparison, and Outcome (PICO) structure is detailed in Table 1. This study has been registered in PROSPERO (International Prospective Register of Systematic Reviews) [33], with registration number CRD42024585686. The review protocol is available in the PROSPERO record.

**Table 1:** Population, Intervention, Comparison, and Outcome (PICO) framework of wearable device interventions on gait, balance, and quality of life in patients with Parkinson disease under the International Classification of Functioning, Disability, and Health (ICF) framework.

Population and intervention			Comparison		Outcome
**Patients with Parkinson disease**					
	Intervention settings	Comparison between the wearable device group and the control group		Gait performance (10MWT^a^, Stride length, DST^b^, FOG-Q^c^, UPDRS III^d^)	
**Hoehn and Yahr stages 1-3**					
	Hospital	—^e^		d450 Walking	
	Home and community	—		d450 Walking	
	Laboratory	—		b730 Muscle power functions	
	Intervention personnel	—		Balance function (BBSf, Mini-BESTestg)	
	Physiotherapist	—		b235 Vestibular functions	
	Clinical researchers	—		d410 Changing basic body position	
	Professional instructors Interventions	—		Quality of life (PDQh, EQ-5D-utility, FES-Ii)	
	Use of wearable devices	—		b130 Energy and drive functions	
	Conventional rehabilitation	—		d920 Recreation and leisure	
	Intervention protocol	—		d570 Looking after one’s health	
	Type	—		d760 Family relationships	
	Frequency	—		—	
	Duration	—		—	

^a^10MWT: 10-Meter Walk Test.

^b^DST: double support time.

^c^FOG-Q: Freezing of Gait Questionnaire.

^d^UPDRS III: Unified Parkinson’s Disease Rating Scale Part III.

^e^Not applicable.

^f^BBS: Berg Balance Scale.

^g^Mini-BESTest: Mini Balance Evaluation Systems Test.

^h^PDQ: Parkinson Disease Questionnaire.

^i^FES-I: Falls Efficacy Scale–International.

### Information Sources

We conducted a comprehensive literature search in PubMed (National Library of Medicine), Embase (Elsevier, Embase.com), the Cochrane Library (Wiley), and Web of Science Core Collection (Clarivate) from inception to November 18, 2025. We also searched ClinicalTrials.gov on November 18, 2025, to identify ongoing or unpublished RCTs evaluating wearable-device interventions in PD.

Databases were searched separately via their native interfaces (ie, no multidatabase searching on a single platform). No search updates were performed after the final search date, and no email alerts were set. No additional online or print sources (eg, journal tables of contents, conference proceedings, or websites) were purposefully searched or browsed.

We screened the reference lists of relevant systematic reviews and all included trials (backward citation searching) to identify additional eligible studies. Reference list screening was performed during full-text assessment and was completed on November 18, 2025. We did not perform forward citation tracking (citing-reference searches) or set up citation alerts. When necessary, we contacted corresponding authors of included studies to request clarification or missing outcome data. No additional information sources or search methods (eg, gray literature databases, preprint servers, or Google Scholar) were used beyond those described above.

### Search Strategy

The search strategy combined controlled vocabulary terms (eg, MeSH [Medical Subject Headings] and Emtree) and free-text keywords related to PD, wearable or sensor-based devices, and RCTs. We did not use published search filters (eg, validated RCT filters); trial design was captured using a combination of controlled vocabulary and free-text terms. Search strategies were developed de novo and were not adapted or reused from previous literature reviews.

Searches were limited to English-language publications involving human adults; no restrictions were placed on publication status. These restrictions were applied to align with the eligibility criteria (adult PD populations and RCTs evaluating intervention efficacy) and for feasibility of screening and data extraction.

The search strategy and its reporting followed PRISMA-S (Preferred Reporting Items for Systematic Reviews and Meta-Analyses literature search extension). The full search strings for each database and information source (including all search terms, Boolean operators, limits, and the date last searched) are provided in S1 in Multimedia Appendix 1. The search strategy was not formally peer-reviewed.

### Eligibility Criteria

#### Inclusion Criteria

Studies were eligible if they met all of the following criteria:

(1) Study design: RCTs evaluating wearable-device interventions in people with PD.

(2) Participants: adults (≥18 years) with a clinical diagnosis of PD based on internationally accepted criteria. Participants were required to be clinically stable; where pharmacotherapy was used (eg, levodopa or other anti-Parkinsonian medications), medication regimens were stable during the trial period (ie, no major medication changes). No restrictions were applied with respect to sex.

(3) Interventions and comparators: the experimental group received a wearable-device intervention (eg, wearable cueing or feedback or sensor-based device, assisted rehabilitation), either as a standalone intervention or as an adjunct to usual care (eg, conventional training and/or pharmacotherapy). Control conditions included usual care without an active wearable-device component and could include conventional training and/or pharmacotherapy, with or without sham or placebo wearable devices.

(4) Outcomes: studies were eligible if they reported at least one prespecified outcome in any of the following domains: gait and mobility, motor severity, balance, and health-related quality of life outcomes. Detailed outcome definitions and measurement instruments are provided in Section 2.6 (Data items—outcomes).

#### Exclusion Criteria

Studies were excluded if they:

(1) Were not RCTs (eg, nonrandomized studies, observational designs, case series, and qualitative studies);

(2) Enrolled participants younger than 18 years or did not clearly involve a PD population;

(3) Did not evaluate an eligible wearable-device intervention and/or lacked an appropriate control condition;

(4) Did not report any outcomes of interest and/or did not provide sufficient data for extraction (eg, missing summary statistics), and the necessary data could not be obtained from the report or study authors; or

(5) Had unavailable full texts.

For duplicate publications or multiple reports from the same trial, the most complete or most recent report was retained, and companion reports were used to supplement missing details where applicable.

### Selection Process

Two reviewers (JW and WZ) independently screened all retrieved records. In the first stage, titles and abstracts were assessed for relevance and study design. In the second stage, full texts were evaluated against the predefined inclusion and exclusion criteria. Any discrepancies were resolved by discussion; if consensus could not be reached, a third reviewer adjudicated. The study selection process was documented using a PRISMA 2020 flow diagram. The completed PRISMA checklist is provided in Multimedia Appendix 2. No automation tools were used for study selection. As only English-language reports were eligible, no translation was required for screening.

### Data Collection Process

Data were extracted independently by 2 reviewers (MF and NX) using a piloted, standardized extraction form. Discrepancies in data extraction were resolved by discussion, with adjudication by a third reviewer when necessary. For each prespecified outcome, we extracted data at baseline and at the end of the intervention (postintervention values and/or change scores, as reported), prioritizing the end-of-intervention assessment for the primary synthesis. Risk-of-bias information for each included study was extracted in parallel to support risk-of-bias assessments and GRADE judgments. When required data were missing or unclear, attempts were made to contact study authors; otherwise, available data were analyzed as reported. No automation tools were used for data extraction. As only English-language reports were eligible, no translation was required for data collection.

### Data Items (Outcomes)

Outcome data were extracted for the prespecified outcome domains and measurement instruments, with scale directions checked to ensure consistent interpretation. Outcomes of interest included:

1. Gait and mobility: 10-Meter Walk Test (10MWT) time, stride length, DST, and freezing of gait assessed by the Freezing of Gait Questionnaire (FOG-Q) or New Freezing of Gait Questionnaire (NFOG-Q).
2. Motor severity: UPDRS III.
3. Balance: Berg Balance Scale (BBS), Mini Balance Evaluation Systems Test (Mini-BESTest), and Performance-Oriented Mobility Assessment—Balance subscale (POMA balance).
4. Health-related quality of life outcomes: Parkinson Disease Questionnaire (PDQ-39 or PDQ-8), EQ-5D utility index, and Falls Efficacy Scale-International (FES-I).

For each outcome, we extracted the assessment time points and baseline and end-of-intervention data (postintervention values and/or change scores as reported), including the mean and SD or SE and sample size per group.

### Data Items (Other Variables)

The following additional data items were extracted:

1. Study characteristics: first author, publication year, country, study design (restricted to RCTs), recruitment source, sample size per arm, follow-up duration, trial registration, ethics approval, funding sources, and declared conflicts of interest.
2. Participant baseline characteristics: age, sex distribution, disease duration, PD severity or stage, medication status (ON or OFF or stable medication), and baseline comparability between groups.
3. Intervention and comparator details: type and modality of wearable device (eg, auditory, visual or vibrotactile cueing, feedback features, stimulation system, and sensor placement), training context (clinic, home, or laboratory), intervention dose (frequency, session duration, or total intervention period), cointerventions, adherence, compliance, dropouts, and adverse events.

### Study Risk of Bias Assessment

Risk of bias was assessed at the study level for included RCTs using the Cochrane Risk of Bias tool (RoB 1), following the Cochrane Handbook (version 5.1.0). The following domains were evaluated, including random sequence generation, allocation concealment, blinding of participants and personnel, blinding of outcome assessment, incomplete outcome data, selective reporting, and other sources of bias. Each domain was judged as low, high, or unclear risk of bias. An overall risk-of-bias judgment was derived as follows: studies were rated low overall if all domains were low risk, high overall if one or more domains were high risk, and unclear overall otherwise. Two reviewers (JW and WZ) independently performed the assessments, and disagreements were resolved through discussion, with adjudication by a third reviewer (QZ) when necessary. Where required, study authors were contacted to clarify information relevant to risk-of-bias judgments. No automation tools were used for risk-of-bias assessment.

### Effect Measures

All prespecified outcomes were continuous measures. For outcomes assessed using the same instrument and reported on the same scale across studies, we synthesized effects as mean differences (MDs) with 95% CIs (eg, 10MWT time [seconds], stride length [meters], double support time [% gait cycle], UPDRS III [points], EQ-5D utility, FES-I [points]). When the same construct was assessed using different instruments, we used standardized mean differences (SMDs; Hedges *g*) with 95% CIs (eg, pooling balance outcomes across BBS, Mini-BESTest, and POMA balance; pooling quality-of-life outcomes across PDQ-39 and PDQ-8). Effect directions were checked before synthesis to ensure consistent interpretation across scales (eg, lower scores indicating improvement for PDQ, FOG-Q and NFOG-Q, UPDRS III, and 10MWT time; higher scores indicating improvement for balance scales).

For each outcome, we prioritized end-of-intervention assessments. We preferentially extracted post-intervention values; when only change-from-baseline data were reported, these were used as reported. Where change scores were not reported but could be derived from available pre- and postintervention summary statistics, they were calculated using standard formulas; otherwise, postintervention values were used. For all meta-analyses, effect estimates were presented with 95% CIs and, when 3 or more studies were available, 95% prediction intervals. We did not prespecify universal thresholds for “small, moderate, or large” effects; interpretation emphasized clinical relevance on the original scale (for MDs) and certainty of evidence (GRADE).

### Synthesis Methods

#### Eligibility for Each Synthesis

For each prespecified outcome, studies were eligible for quantitative synthesis if they were randomized controlled trials and reported the relevant outcome with extractable data at baseline and at the end of the intervention for at least one wearable-device versus control comparison. When fewer than 2 studies were available for a given outcome, results were summarized narratively without pooling.

#### Preparing for Synthesis

Prior to synthesis, outcome definitions, units, and scale directions were checked to ensure consistent interpretation of effect estimates (eg, higher scores consistently indicated better or worse status as appropriate). Where required summary statistics were not directly reported, we attempted to derive them from available information (eg, SEs or 95% CIs) using standard formulas; when this was not possible, study authors were contacted. If key statistics remained unavailable after these attempts, the outcome was not pooled.

#### Methods Used to Tabulate and Visually Display Results

Study characteristics and extracted outcome data were summarized in tables. Meta-analysis results were presented using forest plots. Risk-of-bias judgments were summarized both graphically (eg, traffic-light plots) and in tabular form.

#### Methods Used to Synthesize Results

Studies were grouped for synthesis by outcome domain and measurement instrument. We conducted separate meta-analyses for (1) gait performance (10MWT, stride length, double support time, FOG-Q, and NFOG-Q), (2) motor severity (UPDRS III), (3) balance (BBS, Mini-BESTest, and POMA balance), and (4) health-related quality of life outcomes (PDQ-39, PDQ-8, EQ-5D utility, and FES-I). When multiple time points were reported, we prioritized the end-of-intervention assessment for the primary synthesis.

Meta-analyses were conducted in R (meta package; R Core Team). For continuous outcomes, pooled effects were expressed as MD when studies used the same measurement scale or SMD (Hedges *g*) when different instruments were used to assess the same construct, both with 95% CIs. Given expected clinical and methodological variability across interventions, all meta-analyses used a random-effects model [34]. Between-study variance (τ²) was estimated using restricted maximum likelihood (REML) [35], and the Hartung-Knapp-Sidik-Jonkman method was applied to obtain more robust CIs [36]. Statistical heterogeneity was quantified using *I*² and τ/τ² statistics. Where at least three studies were available, 95% prediction intervals were calculated using the bootstrap approach proposed by Nagashima et al [37], (method.predict=“NNF” in the meta package; requires the pimeta package) to improve performance in meta-analyses with few studies [38].

#### Methods Used to Explore Possible Causes of Heterogeneity

Formal investigations of heterogeneity (eg, subgroup analyses, meta-regression, or statistical tests for interaction) were not undertaken because most syntheses included few studies and statistical heterogeneity was generally low, making effect-modifier analyses unreliable.

#### Sensitivity Analyses

To examine robustness to measurement heterogeneity, prespecified sensitivity analyses were conducted when feasible (typically when 3 or more studies were available) by restricting analyses to studies using the same instrument for a given construct (eg, pooling FOG-Q only when most studies used FOG-Q rather than NFOG-Q; pooling BBS only when multiple balance instruments were used). Sensitivity analysis results were reported alongside the primary analyses.

#### Reporting Bias Assessment

Because the number of included studies contributing to each synthesis was small, formal assessment of reporting bias due to missing results (eg, funnel plot asymmetry and Egger regression test for small-study effects) was planned only when 10 or more studies were available for a given outcome. When fewer than 10 studies were available, formal statistical and graphical assessments were not undertaken because of limited power and interpretability. In such cases, the potential impact of missing results was considered qualitatively when judging certainty of evidence within the GRADE framework (publication bias domain).

#### Certainty Assessment

The certainty (confidence) in the body of evidence for each prespecified outcome was assessed using the GRADE approach, considering five domains: risk of bias, inconsistency, imprecision, indirectness, and publication bias [39]. Evidence certainty was categorized as high, moderate, low, or very low. Two reviewers (JW and WZ) independently performed the GRADE assessments, and disagreements were resolved by discussion, with adjudication by a third reviewer (QZ) when necessary.

## Results

### Study Selection

**Figure 1:**
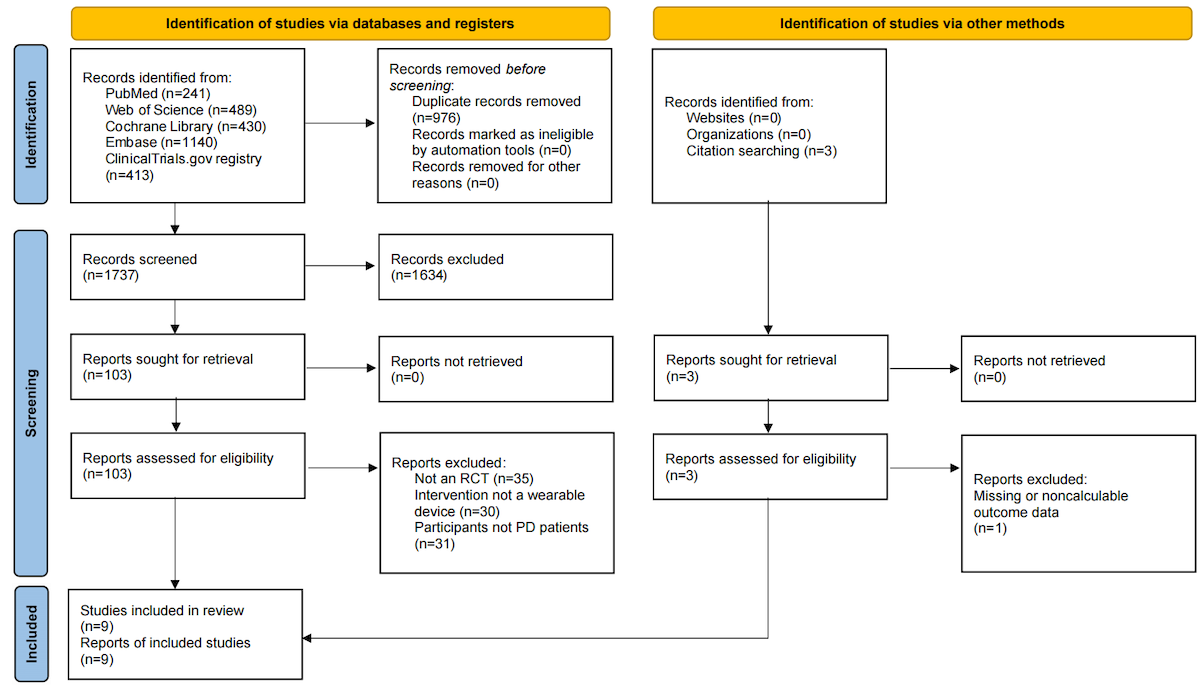
PRISMA (Preferred Reporting Items for Systematic Reviews and Meta-Analyses) 2020 flow diagram of study selection.

A total of 2300 relevant articles were identified. After the initial screening, 866 duplicate articles were excluded. Based on the titles and abstracts, a further 1394 articles were excluded. Following a full-text review according to the inclusion and exclusion criteria, 31 additional articles were excluded. Ultimately, 9 studies were included in the analysis [25,40-47]. The literature screening process is shown in Figure 1. Full-text reports that appeared potentially eligible but were excluded, together with reasons for exclusion, are listed in S2 in Multimedia Appendix 1.

### Risk of Bias in Studies

**Figure 2:**
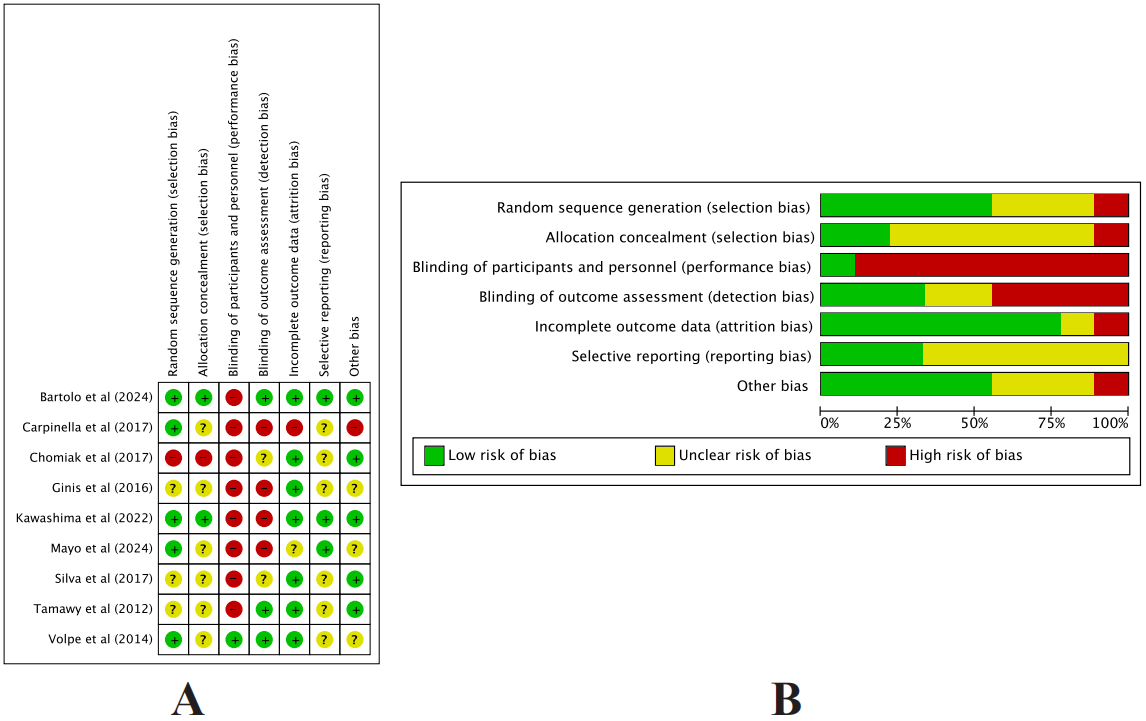
Quality assessment of included studies. (A) Risk of bias summary. (B) Risk of bias graph: percentage summary across all included studies for each type of bias [25,40-46,48].

Figures 2A and 2B summarize the risk-of-bias assessment [25,40-47]. Overall, methodological quality was mixed. Most trials reported adequate random sequence generation, whereas details of allocation concealment were frequently insufficient or not reported, leading to many domains being judged as having an unclear risk of bias. Because blinding of participants and personnel was largely not feasible given the nature of wearable-device interventions, several studies were rated at high risk of performance bias. In contrast, blinding of outcome assessors was implemented in approximately half of the trials. Incomplete outcome data and selective reporting were generally judged to be at low risk of bias, with most studies reporting attrition and prespecified outcomes adequately. Other potential sources of bias were usually rated as unclear.

### Study Characteristics

A total of 9 studies were included [25,40-47], all of which were published in English, involving a total of 260 PD patients. In most studies, the average age of patients was over 60 years, and the average disease duration exceeded 3 years. Two studies [42,45] conducted 10MWT analysis, 4 studies [40,41,43,46] analyzed stride length, 3 studies [41,43,46] analyzed DST, 4 studies [41,42,44,45] analyzed FOG-Q, 5 studies [41,42,45-47] analyzed UPDRS III, 5 studies [41,42,45-47] analyzed balance function, 3 studies [42,45,47] analyzed PDQ-39, 2 studies [25,45] analyzed EQ-5D utility, and 2 studies [41,44] analyzed FES-I. In most of these studies, patients maintained stable medication regimens before and during the treatment period. Detailed information on the patients and wearable device variables used in these studies is shown in Table 2.

**Table 2:** Characteristics of included studies

Included studies	T^a^/C^b^ (n)	Age in years (T/C), mean (SD)	Hoehn and Yahr stage (T/C)	Disease duration (T/C) years	Wearable prompt device	Feedback characteristics	Stimulation system	Intervention method, (T/C)	Training environment	Intervention duration	Outcome measures
El-Tamawy et al (2012) [40]	15/15	61.4 (7.28)/63.2 (5.6)	2.8 (0.5)/2.6 (0.4)	4.0 (0.9)/3.8 (0.9)	Vibration device placed in the shoes	Proprioception	Open-loop system	Proprioceptive neuromuscular facilitation technique and vibration stimulation + physical therapy program / conventional physical therapy	Laboratory	8 weeks, 3 times/week, 45 minutes/session	Stride length
Volpe et al (2014) [47]	20/20	66.5 / 69.	3.0 / 3.0	6.0 / 6.5	Equistasi nanotech device (Equistasi Srl) placed on C7 and both soleus tendons	Proprioception	Open-loop system	Proprioceptive stimulation (Equistasi) + balance training / balance training only	Laboratory setting (hospital)	8 weeks, 5 times/week, 60 minutes/session	UPDRS III (Unified Parkinson Disease Rating Scale Part III); DST (double support time); BBS (Berg Balance Scale); PDQ (Parkinson Disease Questionnaire)
Ginis et al (2016) [41]	20/18	67.3 (8.13)/ 66.11 (8.07)	2.3 (0.44)/2.2 (0.39)	10.65 (5.39)/11.67 (7.63)	CuPiD system	Auditory feedback	Open-loop system	Real-time gait feedback training provided by the CuPiD system / personalized gait advice	Home	6 weeks, 3 times/week, 30 minutes/session	Stride length; UPDRS III (Unified Parkinson Disease Rating Scale Part III); FOG-Q (Freezing of Gait Questionnaire); DST (double support time); FES-I (Falls Efficacy Scale - International); Mini-BESTest (Mini Balance Evaluation Systems Test)
Carpinella et al (2017) [42]	17/20	73 (7.1)/ 75.6 (8.2)	2.7 (0.7)/2.9 (0.5)	7.5 (3.2)/ 10.3 (5.7)	Gamepad system	Visual and auditory feedback	Open-loop system	Gait and balance training using the Gamepad system / personalized physical therapy	Hospital	20 sessions, 3 times/week, 45 minutes/session	10MWT (10-Meter Walk Test); UPDRS III (Unified Parkinson Disease Rating Scale Part III); FOG-Q (Freezing of Gait Questionnaire); BBS (Berg Balance Scale); PDQ (Parkinson Disease Questionnaire)
Lirani-Silva et al (2017) [43]	10/9	70.4 (6.87)/72 (6.2)	2 (0.5)/ 1.9 (0.4)	N/A^c^	Textured insoles	Proprioceptive feedback	Open-loop system	Using textured insoles / using conventional insoles	Home	1 week	Stride length; DST (double support time)
Chomiak et al (2017) [44]	5/6	70.8 (5.6)/ 69 (5.7)	2.5 (0.50/2.7 (0.41)	15.4 (5.4)/ 11.2 (5)	Ambulosono platform	Auditory feedback	Closed-loop system	Feedback through music playback / Feedback through CBC podcasts	Home	4 weeks, 3 times/week, 10-20 minutes/session	FOG-Q (Freezing of Gait Questionnaire); FES-I (Falls Efficacy Scale - International)
Kawashima et al (2022) [45]	5/7	76.6 (5.3)/ 75.4 (5.7)	2.4 (0.55)/2.4 (0.79)	11.2 (5.8)/ 12.4 (4.6)	Step Management Assist exoskeleton	Proprioceptive feedback	Open-loop + closed-loop system	Gait training using the SMA / conventional gait training	Home-based	3 months, 10 sessions, 30 minutes per session	10MWT (10-Meter Walk Test); stride length; UPDRS III (Unified Parkinson Disease Rating Scale Part III); FOG-Q (Freezing of Gait Questionnaire); BBS (Berg Balance Scale); PDQ (Parkinson Disease Questionnaire); EQ-5D-utility
Bartolo et al (2024) [46]	26/26	73.0 (7.3)/ 70.3 (11)	2 (0.6)/2.1 (0.7)	9.4 (3.1)/ 9.8 (3.9)	Q-Walk system	Visual feedback	Closed-loop system	Gait training using the Q-Walk system / conventional gait training	Clinical rehabilitation	2 weeks, 5 sessions per week, 90 minutes per session	10MWT (10-Meter Walk Test); UPDRS III (Unified Parkinson Disease Rating Scale Part III); DST (double support time); POMA Balance (Performance-Oriented Mobility Assessment Balance Subscale)
Mayo et al (2024) [25]	14/7	70.2 (8.5)/ 70.7 (8.8)	2～3	N/A	Heel2Toe sensor	Auditory feedback	Closed-loop system	Training with Heel2Toe sensor / training according to the manual	Home-based	3 months, twice daily, 5 minutes per session	PDQ (Parkinson Disease Questionnaire); EQ-5D-utility

^a^T: trials (experimental group).

^b^C: controls (control group).

^c^N/A: not applicable.

### Results of Syntheses

#### Gait Performance

##### 10MWT

**Figure 3:**
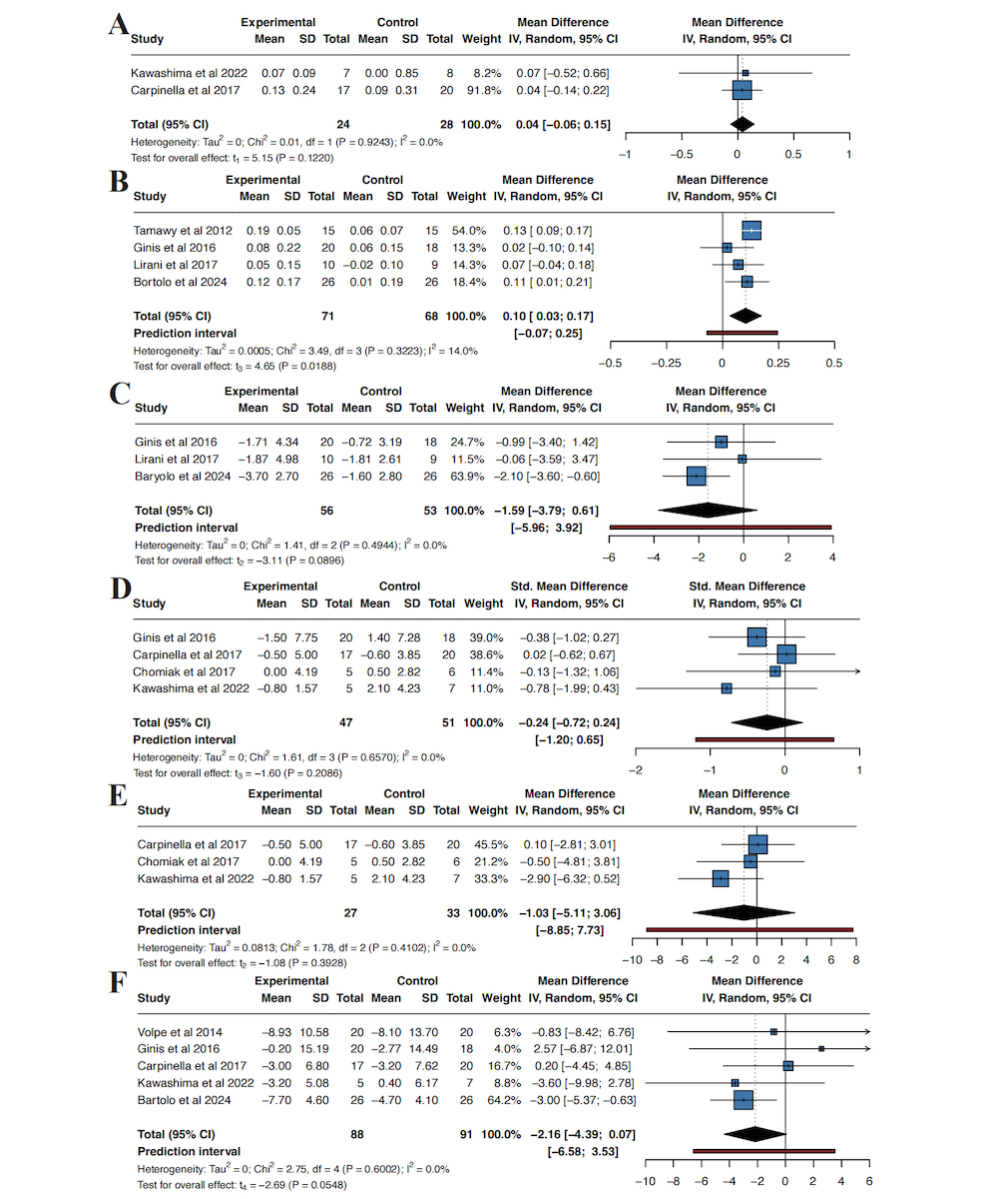
Meta-analyses of gait performance and motor severity outcomes for wearable-device interventions versus control in Parkinson disease. (A) 10-Meter Walk Test (10MWT) time (seconds). (B) Stride length (meters). (C) Double support time (DST, % gait cycle). (D) Freezing of gait, pooled across the Freezing of Gait Questionnaire (FOG-Q) and the New Freezing of Gait Questionnaire (NFOG-Q), expressed as standardized mean differences (SMDs). (E) Sensitivity analysis for freezing of gait restricted to studies using the FOG-Q only, expressed as mean differences (points). (F) Unified Parkinson Disease Rating Scale Part III (UPDRS III) score (points) [40-46,48].

Two studies [42,45] evaluated the 10MWT outcome. Between-study heterogeneity was negligible (τ² 0, τ=0; *I*²=0%; Q test *P*=.92). The pooled analysis showed no clear evidence of a difference between the wearable-device and control groups in 10MWT (MD 0.04, 95% CI –0.06 to 0.15; *P*=.12; Figure 3A). Prediction intervals were not calculated because fewer than 3 studies were available.

##### Stride Length

Four studies [40,41,43,46] evaluated stride length (wearable-device group n=71; control group n=68). Between-study heterogeneity was low (τ²=0.0005; τ=0.02; *I*²=14.0%; Q test *P*=.32). Based on our prespecified random-effects model (REML with Hartung–Knapp adjustment), wearable-device interventions were associated with a small but statistically significant improvement on average in stride length compared with controls (MD 0.10 m, 95% CI 0.03-0.17; *P*=.02). The 95% prediction interval was –0.07 to 0.25 m (Figure 3B).

##### DST

Three studies [41,43,46] evaluated DST (wearable-device group n=56; control group n=53). Between-study heterogeneity was negligible (τ²=0; τ=0; *I*²=0.0%; Q test *P*=.49). Based on our prespecified random-effects model (REML with Hartung–Knapp adjustment), the pooled analysis showed no clear evidence of a difference in DST between wearable-device interventions and controls (MD –1.59% gait cycle, 95% CI –3.79 to 0.61; *P*=.09). The 95% prediction interval ranged from –5.96 to 3.92% gait cycle (Figure 3C).

##### FOG-Q

Four studies [41,42,44,45] evaluated freezing of gait using either the FOG-Q or NFOG-Q (wearable-device group n=47; control group n=51). Between-study heterogeneity was negligible (τ²=0; τ=0; *I*²=0.0%; Q test *P*=.66). Using our prespecified random-effects model (REML with Hartung–Knapp adjustment), the pooled analysis showed no clear evidence of a difference in freezing of gait between wearable-device interventions and controls (SMD –0.24, 95% CI –0.72 to 0.24; *P*=.21). The 95% prediction interval was –1.20 to 0.65 (Figure 3D). In sensitivity analyses restricted to studies using the same instrument (FOG-Q only; 3 studies [42,44,45], wearable-device group n=27; control group n=33), heterogeneity remained low (τ²=0.0813; τ=0.29; *I*²=0.0%; Q test *P*=.41). The pooled effect again showed no statistically significant between-group difference (MD –1.03 points, 95% CI –5.11 to 3.06; *P*=.39). The 95% prediction interval ranged from –8.85 to 7.73 points (Figure 3E).

##### UPDRS III

Five studies [41,42,45-47] evaluated UPDRS III (wearable-device group n=88; control group n=91). Between-study heterogeneity was negligible (τ²=0; τ=0; *I*²=0.0%; Q test *P*=.60). Using the prespecified random-effects model (REML with Hartung–Knapp adjustment), the pooled analysis found no statistically significant difference in UPDRS III between wearable-device interventions and controls (MD –2.16 points, 95% CI –4.39 to 0.07; *P*=.06). The 95% prediction interval was –6.85 to 3.53 points (Figure 3F).

#### Balance Function

**Figure 4:**
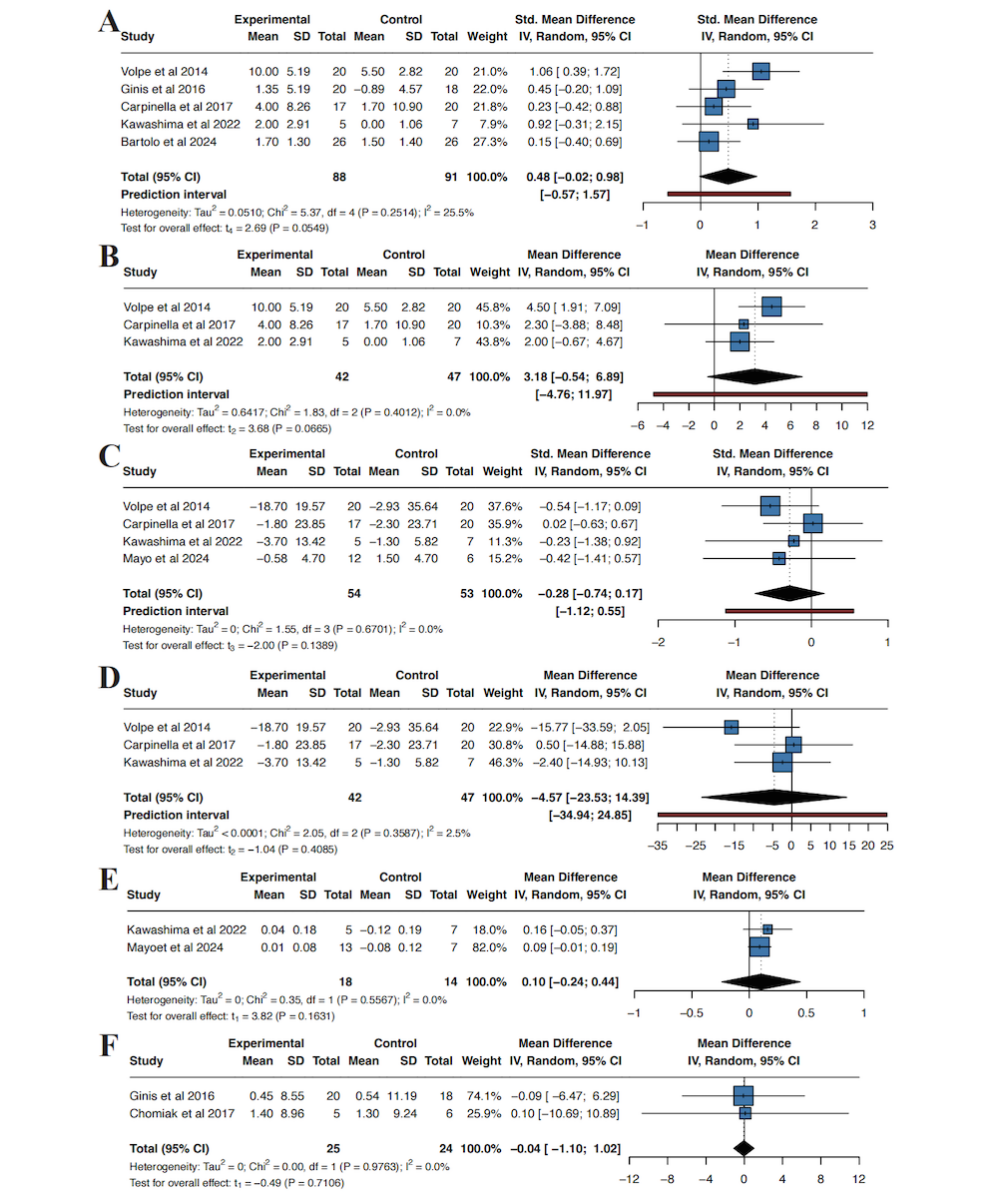
Meta-analyses of balance, health-related quality of life, and fear-of-falling outcomes for wearable-device interventions versus control in Parkinson disease. (A) Balance performance expressed as standardized mean differences (SMDs), pooling the Berg Balance Scale (BBS), Mini Balance Evaluation Systems Test (Mini-BESTest), and the balance subscale of the Performance-Oriented Mobility Assessment (POMA balance). (B) Sensitivity analysis for balance restricted to studies using the BBS only, expressed as mean differences (MDs, points). (C) Health-related quality of life assessed with Parkinson Disease Questionnaire (PDQ) instruments, pooling PDQ-39 and PDQ-8 as SMDs. (D) Sensitivity analysis for PDQ restricted to PDQ-39 only, expressed as MDs (points). (E) EQ-5D utility index scores. (F) Fear of falling assessed with the Falls Efficacy Scale–International (FES-I, points) [25,41,42,44-46,48].

Three studies [42,45,47] reported results using the BBS, one study [41] reported using the Mini-BESTest, and one study [46] reported using the POMA balance (wearable-device group n=88; control group n=91). Because different scales were used to measure the same construct, results were pooled using SMD. Between-study heterogeneity was low (τ²=0.0510; *I*²=25.5%; Q test *P*=.25). Using a random-effects model (REML with Hartung–Knapp adjustment), the pooled analysis showed no statistically significant difference in balance between wearable-device interventions and controls (SMD 0.48, 95% CI –0.02 to 0.98; *P*=.06). The 95% prediction interval ranged from –0.57 to 1.57 (Figure 4A).

To examine the robustness of this SMD result to measurement heterogeneity, we performed a sensitivity analysis restricted to the three studies using the same balance instrument (BBS; wearable-device group n=42; control group n=47). Heterogeneity remained negligible (τ²=0.6417; *I*²=0%; Q test *P*=.40). The pooled effect was not statistically significant (MD 3.18 BBS points, 95% CI –0.54 to 6.89; *P*=.07). The 95% prediction interval ranged from –4.76 to 11.97 BBS points (Figure 4B).

#### Quality of Life

##### PDQ

Four studies [25,42,45,47] assessed quality of life using PDQ instruments. Three studies used the PDQ-39 [42,45,47], whereas 1 study [25] used the PDQ-8 (wearable-device group n=54; control group n=53). Therefore, the primary meta-analysis pooled these outcomes using SMD (Hedges *g*). Between-study heterogeneity was negligible (τ²=0; τ=0; *I*²=0%; Q test *P*=.67). Based on our prespecified random-effects model (REML with Hartung–Knapp adjustment), the pooled analysis showed no statistically significant difference in PDQ scores between wearable-device interventions and controls (SMD –0.28, 95% CI –0.74 to 0.17; *P*=.14). The 95% prediction interval ranged from –1.12 to 0.55 (Figure 4C). To examine the robustness of the pooled effect to measurement heterogeneity, we repeated the analysis after excluding the PDQ-8 study and pooling only PDQ-39 outcomes using MD (wearable-device group n=42; control group n=47). Heterogeneity remained low (τ²<0.0001; *I*²=2.5%; Q test *P*=.36). The pooled result showed no statistically significant difference between groups (MD –4.57, 95% CI –23.53 to 14.39; *P*=.41). The 95% prediction interval was –34.94 to 24.85 (Figure 4D).

##### EQ-5D Utility

Two studies [25,45] evaluated EQ-5D utility (wearable-device group n=18; control group n=14). Between-study heterogeneity was negligible (τ²=0; τ=0; *I*²=0.0%; Q test *P*=.56). Using the prespecified random-effects model (REML with Hartung–Knapp adjustment), the pooled analysis showed no statistically significant difference in EQ-5D utility between wearable-device interventions and controls (MD 0.10, 95% CI –0.24 to 0.44; *P*=.16; Figure 4E). Prediction intervals were not calculated because fewer than three studies were available.

##### FES-I

Two studies [41,44] evaluated fear of falling using the FES-I (wearable-device group n=25; control group n=24). Between-study heterogeneity was negligible (τ²=0; τ=0; *I*²=0.0%; Q test *P*=.98). Using the prespecified random-effects model (REML with Hartung–Knapp adjustment), the pooled analysis showed no statistically significant difference in FES-I scores between wearable-device interventions and controls (MD –0.04 points, 95% CI –1.10 to 1.02; *P*=.71; Figure 4F). Prediction intervals were not calculated because fewer than three studies were available.

### Certainty of Evidence

According to GRADE, the overall certainty of evidence ranged from low to very low for all outcomes. For stride length, the certainty was rated as low, reflecting consistent direction of effect across four randomized trials and reasonably precise estimates, but downgrading for very serious risk of bias related to unclear allocation concealment, lack of blinding of outcome assessors, and incomplete outcome data. For all other prespecified outcomes—including 10MWT, DST, freezing of gait, UPDRS III, balance scales, PDQ, EQ-5D utility, and FES-I—the certainty of evidence was judged very low. These ratings were primarily driven by very serious risk of bias and very serious imprecision, with small sample sizes per outcome and wide confidence and prediction intervals that encompassed both no effect and potentially important benefit or harm. Inconsistency and indirectness were generally not considered serious (Table 3). Because the number of included studies was small, we could not formally evaluate funnel plot asymmetry; therefore, the possibility of small-study effects could not be ruled out [48].

**Table 3:** Certainty of evidence assessment of included studies.

Certainty assessment								Number of patients			Effect					
Number of studies	Study design	Risk of bias	Inconsistency	Indirectness	Imprecision	Other considerations	Intervention		Comparison	Relative (95% CI)		Absolute (95% CI)	Certainty		Importance	
10-Meter Walk Test																
2	Randomized trials	Very serious^a^	Not serious	Not serious	Very serious^b^	None	24		28	—^c^		MD^d^ 0.04s (95% CI –0.06 to 0.15)	Very low^a,^^b^		Important	
Stride length																
4	Randomized trials	Very serious^a^	Not serious	Not serious	Not serious	None	71		68	—		MD 0.10 m, (95% CI 0.03-0.17)	Low^a^		Important	
Double support time																
3	Randomized trials	very serious^a^	Not serious	Not serious	Very serious^b^	None	56		53	—		MD 1.59 % gait cycle, (95% CI –3.79 to 0.61)	Very low^a,^^b^		Important	
Freezing of Gait Questionnaire																
4	Randomized trials	Very serious^a^	Not serious	Not serious	Very serious^b^	None	47		51	—		SMD^e^ –0.24, (95% CI –0.72 to 0.24)	Very low^a,^^b^		Important	
Unified Parkinson Disease Rating Scale, Part III																
5	Randomized trials	Very serious^a^	Not serious	Not serious	Serious^b^	None	88		91	—		MD –2.16 points, (95% CI –4.39 to 0.07)	Very low^a,^^b^		Important	
Balance function																
5	Randomized trials	Very serious^a^	Not serious	Not serious	Serious^b^	None	88		91	—		SMD 0.48, (95% CI –0.02 to 0.98)	Very low^a,^^b^		Important	
Parkinson Disease Questionnaire																
4	Randomized trials	Very serious^a^	Not serious	Not serious	Serious^b^	None	55		53	—		SMD –0.28, (95% CI –0.74 to 0.17)	Very low^a,^^b^		Important	
EQ-5D questionnaire																
2	Randomized trials	Very serious^a^	Not serious	Not serious	Very serious^b^	None	18		14	—		MD 0.10, (95% CI –0.24 to 0.44)	Very low^a,^^b^		Important	
Falls Efficacy Scale–International																
2	Randomized trials	Very serious^a^	Not serious	Not serious	Very serious^b^	None	25		24	—		MD –0.04, (95% CI –1.10 to 1.02)	Very low^a,^^b^		Important	

^a^Risk of bias: downgraded 1 level when most contributing trials had unclear or high risk of bias in one or more key domains (eg, allocation concealment, blinding of outcome assessment, incomplete outcome data); downgraded 2 levels when the pooled evidence was dominated by trials at high risk of bias across multiple key domains.

^b^Imprecision: downgraded 1 level when the total information size was small and the 95% CI included the null effect; downgraded 2 levels when the total information size was very small and the 95% CI was wide, spanning both clinically important benefit and no effect (or harm).

^c^not applicable.

^d^MD: mean difference.

^e^SMD: standardized mean difference.

## Discussion

### Interpretation

This systematic review and meta-analysis synthesized RCTs evaluating the effects of wearable-device interventions on gait, balance, and quality of life in people with PD, interpreting the findings within the ICF framework [49]. We found a small but statistically significant improvement on average in stride length; however, most other outcomes—including short-distance gait speed, double support time, freezing of gait, balance scales, health-related quality of life, and fear of falling—did not show clear between-group differences. These results suggest that current wearable technologies may have only modest effects on specific gait parameters at the level of body functions and activities, with insufficient evidence for consistent improvement in balance or overall quality of life. Importantly, prediction intervals for several outcomes crossed the null, indicating that effects may vary across populations and implementation contexts and may be minimal or absent in some settings.

Regarding gait and motor outcomes, wearable devices produced a small improvement on average in stride length with low statistical heterogeneity, although the prediction interval suggests that effects may be minimal in some settings, consistent with the idea that cueing and feedback can help increase step amplitude in PD. In contrast, we did not observe clear between-group differences in 10-meter walk performance, double support time, freezing-of-gait scores, or UPDRS III, and the corresponding prediction intervals generally spanned both small benefits and no effect. Overall, these results point to at most modest and uncertain benefits of wearable-device interventions on gait and motor severity. Our findings on short-distance gait performance differ from those of a previous meta-analysis by Zhang et al [30], which reported a statistically significant improvement in gait speed with wearable cueing devices. One likely explanation is that Zhang et al [30] pooled several conceptually different outcomes (10MWT, 6-minute walk test, and treadmill-based speed measures) under a single “gait velocity” construct, whereas we analyzed the 10-Meter Walk Test separately and did not combine it with endurance or treadmill outcomes [30]. In addition, we used a more conservative random-effects approach (REML with Hartung–Knapp adjustment and prediction intervals [36]), which yields wider uncertainty intervals and may partly explain why the apparent speed benefit did not remain statistically significant or robust across settings in our analyses. The small but statistically significant improvement in stride length, in the absence of clear effects on 10MWT or freezing measures, may reflect that step amplitude is more directly modulated by external cueing and feedback than more complex phenomena such as gait initiation, freezing episodes, or sustained walking speed; moreover, many participants were in Hoehn and Yahr stages 1-3 with relatively preserved gait, which may limit the observable incremental benefit of wearable devices on standard clinical tests [50].

For balance-related outcomes, this meta-analysis is, to our knowledge, the first to synthesize randomized evidence on the effects of wearable-device interventions on balance function in people with PD. We pooled BBS, Mini-BESTest, and POMA balance subscores using SMDs, as they reflect a common construct of postural control within the ICF domain of body functions and activities. The combined analysis suggested a borderline improvement in balance favoring wearable devices, but the confidence and prediction intervals were wide and included no effect, indicating substantial uncertainty. A key contextual factor is that, among the included trials, only one study explicitly designed the wearable intervention as a balance-focused training program, whereas the others primarily targeted gait initiation or step regulation; thus, the cueing content and training priorities may not have been optimal for producing measurable changes on balance scales. In contrast, a meta-analysis in a nonspecific older-adult population by Mao et al [51] reported clearer balance gains from sensor-based interventions, suggesting that differences in underlying pathology, intervention content, and training dose between PD cohorts and general older adults may be important.

Quality-of-life and fear-of-falling outcomes map more closely to the ICF domains of participation and contextual or personal factors [49]. Across trials, wearable-device interventions did not result in statistically significant improvements in PDQ scores, EQ-5D utility, or FES-I scores, and, where calculated, prediction intervals were wide or included no effect. This pattern suggests that short-term, device-focused interventions may not be sufficient to translate modest improvements in gait or motor scores into perceived gains in overall quality of life or reduced fear of falling. Nonmotor symptoms, psychosocial and environmental factors, and real-world participation constraints were rarely targeted explicitly in the included trials, which may further limit the impact of wearable devices on these broader patient-centered outcomes. From a clinical perspective, these findings underscore that wearable devices are unlikely to replace comprehensive multidisciplinary rehabilitation; rather, they may serve as adjunct tools within a broader program that also addresses cognition, mood, balance confidence, and environmental adaptation [52].

When the findings were interpreted in light of heterogeneity, risk of bias, and GRADE assessments, the overall certainty of evidence for most outcomes was low to very low. Although we restricted inclusion to RCTs, several studies had unclear or high risk of bias in domains such as allocation concealment, blinding of outcome assessment, and incomplete outcome data. Many meta-analyses included only 2-5 trials, leading to wide confidence and, where applicable, prediction intervals that encompassed both no effect and potentially clinically relevant benefit or harm. This imprecision, together with some inconsistency in results and indirectness arising from heterogeneous devices and protocols, resulted in downgrading of GRADE ratings for multiple outcomes [39]. Consequently, our conclusions should be interpreted with caution and considered hypothesis-generating rather than definitive.

### Limitations of Evidence

This study has several limitations that should be considered when interpreting the results. First, the number of eligible trials and the total sample size for each outcome were modest, limiting statistical power and precision, especially for balance, quality-of-life, and fear-of-falling measures. Second, there was substantial diversity in the technical characteristics and application methods of the wearable devices (eg, cueing modality, feedback algorithms, and sensor placement) and in training protocols (setting, frequency, and duration), which may have diluted or masked device-specific effects. Third, most participants were in Hoehn and Yahr stages 1-3, with relatively preserved motor function and independence, restricting the generalizability of our findings to individuals with more advanced disease. Fourth, intervention periods and follow-up durations were relatively short, so the long-term sustainability of any observed benefits remains unknown. Finally, the number of studies per outcome was too small to formally evaluate funnel plot asymmetry; therefore, small-study effects cannot be ruled out.

### Limitations of Review Processes

Several limitations of our review processes should be acknowledged. First, we restricted eligibility to English-language reports, which may have introduced language bias and could have led to missed trials published in other languages. Second, our search did not include gray literature sources (eg, preprint servers, theses, conference proceedings, or general search engines), and we did not perform forward citation tracking; consequently, relevant unpublished or difficult-to-index studies may not have been identified. Third, because all outcomes were informed by fewer than 10 studies, formal assessments of small-study effects (eg, funnel plots and Egger test) were not feasible; therefore, reporting bias due to missing results cannot be excluded. Finally, although we contacted study authors when key information was unclear or missing, incomplete responses may have limited data availability for some syntheses, potentially contributing to imprecision.

### Implications

This review provides an updated and methodologically rigorous synthesis of RCT evidence on wearable devices for gait, balance, and quality of life in Parkinson disease. Innovatively, we integrated the ICF framework with contemporary random-effects meta-analysis (Hartung–Knapp adjustment and prediction intervals) and GRADE to contextualize both the pooled average effects and the expected range of effects across real-world settings. Compared with prior reviews, we avoided pooling conceptually different gait outcomes into a single construct, enabling a more implementation-relevant interpretation of heterogeneity and generalizability. This work contributes to the field by clarifying where evidence is most consistent (eg, stride length) and where effects remain uncertain across settings, particularly for balance and patient-centered outcomes. In real-world practice, wearable technologies are best viewed as adjuncts rather than stand-alone rehabilitation strategies and may need to be integrated with task-specific gait and balance training, behavioral strategies, and environmental modifications to translate modest gait gains into meaningful improvements in confidence and quality of life. Future trials should enroll larger and more diverse populations, extend intervention and follow-up periods, standardize ICF-aligned outcomes, and transparently report device characteristics and implementation strategies to clarify for whom, under what conditions, and how wearables can be optimally integrated into routine care.

### Conclusion

This systematic review and meta-analysis of randomized controlled trials suggests that wearable-device interventions may provide modest benefits on average for specific gait parameters in PD, particularly stride length, but do not consistently improve short-distance gait speed, double support time, freezing of gait, balance performance, health-related quality of life, or fear of falling. Within the ICF framework, current evidence indicates that wearable devices may support selected body functions and activities, yet there is insufficient and uncertain evidence that they reliably enhance broader participation or overall quality of life.

When interpreted alongside heterogeneity estimates, prediction intervals, risk-of-bias assessments, and GRADE ratings, the certainty of evidence for most outcomes is low to very low. Many meta-analyses were based on a small number of heterogeneous trials with modest sample sizes and relatively short intervention and follow-up durations, leading to wide ranges of plausible effects across settings, including minimal or no benefit. Accordingly, our findings should be viewed as cautious and hypothesis-generating rather than definitive and do not currently justify the use of wearable devices as a stand-alone rehabilitation strategy in PD.

From a clinical perspective, wearable technologies are best considered as adjuncts to comprehensive multidisciplinary rehabilitation. Combining wearable cueing and feedback with task-specific gait and balance training, education, and psychosocial and environmental interventions may be required to translate small gains in gait parameters into meaningful improvements in balance confidence, participation, and quality of life. Future trials should recruit larger and more diverse patient populations, including those with more advanced disease; standardize outcome measures across ICF domains; extend intervention and follow-up periods; and transparently report device characteristics and implementation strategies to clarify which patients benefit most and how wearable devices can be optimally integrated into routine care.

### Funding

No external financial support or grants were received from any public, commercial, or not-for-profit entities for the research, authorship, or publication of this article.

### Data Availability

The analytic code used for the meta-analyses and figure generation and the extracted data underlying the analyses are available from the corresponding author upon reasonable request. The data collection forms and additional materials are available upon request.

## Appendix

Multimedia Appendix 1 Full electronic search strategies for all databases and registers, excluded full-text articles with reasons, and PRISMA-S checklists.

Multimedia Appendix 2 The PRISMA 2020 statement.

## Abbreviations

10MWT: 10-Meter Walk Test
BBS: Berg Balance Scale
DST: double support time
FES-I: Falls Efficacy Scale-International
FOG-Q: Freezing of Gait Questionnaire
GRADE: Grading of Recommendations Assessment, Development and Evaluation
ICF: International Classification of Functioning, Disability and Health
IMU: inertial measurement unit
MD: mean difference
MeSH: Medical Subject Headings
Mini-BESTest: Mini Balance Evaluation Systems Test
NFOG-Q: New Freezing of Gait Questionnaire
PD: Parkinson disease
PDQ: Parkinson Disease Questionnaire
PDQ-8: 8-item Parkinson Disease Questionnaire
PDQ-39: 39-item Parkinson Disease Questionnaire
PICO: Population, Intervention, Comparison, and Outcome
POMA balance: Performance-Oriented Mobility Assessment balance subscore
PRISMA: Preferred Reporting Items for Systematic Reviews and Meta-Analyses
PRISMA-S: Preferred Reporting Items for Systematic Reviews and Meta-Analyses literature search extension
PROSPERO: International Prospective Register of Systematic Reviews
RCT: randomized controlled trial
REML: restricted maximum likelihood
RoB: Risk of Bias
SMD: standardized mean difference
UPDRS III: Unified Parkinson Disease Rating Scale Part III
