# Supplementary material for: Effects of Wearable Devices on Parkinson Disease: Systematic Review and Meta-Analysis of Randomized Controlled Trials Within the International Classification of Functioning, Disability, and Health Framework
Source: J Med Internet Res. 2026 Mar 18;28:e85914. doi: 10.2196/85914 (PMC13044512; doi:10.2196/85914)
Supplement: Multimedia Appendix 1 [file jmir_v28i1e85914_app1.doc]

**S1**

| **November 18, 2025** | **Pubmed (241)** |
| --- | --- |
| #1 | "Parkinson Disease"[Mesh]  OR "Parkinsonian Disorders"[Mesh]  OR "Synucleinopathies"[Mesh]  OR parkinson*[tiab]  OR parkinsonian[tiab]  OR parkinsonism[tiab]  OR PD[tiab]  OR "paralysis agitans"[tiab]  OR "Lewy body parkinson*"[tiab] |
| #2 | "Wearable Electronic Devices"[Mesh]  OR "Smartphone"[Mesh]  OR "Cell Phone"[Mesh]  OR "wearable electronic device*"[tiab]  OR "wearable device*"[tiab]  OR "wearable technolog*"[tiab]  OR "wearable sensor*"[tiab]  OR "wearable system*"[tiab]  OR "wearable cue*"[tiab]  OR "body-worn sensor*"[tiab]  OR "body-worn device*"[tiab]  OR "body worn sensor*"[tiab]  OR "body worn device*"[tiab]  OR exoskeleton*[tiab]  OR "robotic exoskeleton*"[tiab]  OR smartphone*[tiab]  OR "smart phone*"[tiab]  OR smartwatch*[tiab]  OR "smart watch*"[tiab]  OR "mobile phone*"[tiab]  OR "activity tracker*"[tiab]  OR "fitness tracker*"[tiab]  OR acceleromet*[tiab]  OR pedomet*[tiab]  OR "inertial sensor*"[tiab]  OR "inertial measurement unit*"[tiab]  OR IMU[tiab]  OR vibrotactile*[tiab]  OR "vibratory stimulation"[tiab]  OR "vibratory stimulus"[tiab]  OR "vibratory device*"[tiab]  OR "textured insole*"[tiab]  OR "shoe insole*"[tiab]  OR Equistasi[tiab]  OR "Equistasi device*"[tiab]  OR "laser shoe*"[tiab]  OR "laser cane*"[tiab]  OR "smart glass*"[tiab]  OR "head-mounted display*"[tiab]  OR HoloLens[tiab]  OR "Google Glass"[tiab] |
| #3 | randomized controlled trial[pt]  OR controlled clinical trial[pt]  OR randomi*ed[tiab]  OR randomly[tiab]  OR randomization[tiab]  OR randomisation[tiab]  OR placebo[tiab]  OR "clinical trials as topic"[Mesh]  OR trial[ti]  NOT (animals[mh] NOT humans[mh]) |
| #4 | #1 AND #2 AND #3 |

| **November 18, 2025** | **Web of Science (489)** |
| --- | --- |
| #1 | TS=( "Parkinson disease"  OR "Parkinson's disease"  OR parkinson*  OR "parkinsonian disorder*"  OR parkinsonism  OR "paralysis agitans"  OR "Lewy body parkinson*") |
| #2 | TS=( "wearable electronic device*"  OR "wearable device*"  OR "wearable technolog*"  OR "wearable sensor*"  OR "wearable system*"  OR "wearable cue*"  OR "body-worn sensor*"  OR "body worn sensor*"  OR "body-worn device*"  OR "body worn device*"  OR exoskeleton*  OR "robotic exoskeleton*"  OR smartphone*  OR "smart phone*"  OR smartwatch*  OR "smart watch*"  OR "mobile phone*"  OR "activity tracker*"  OR "fitness tracker*"  OR acceleromet*  OR pedomet*  OR "inertial sensor*"  OR "inertial measurement unit*"  OR "IMU"  OR vibrotactile*  OR "vibratory stimulation"  OR "vibratory stimulus"  OR "vibratory device*"  OR "textured insole*"  OR "shoe insole*"  OR "Equistasi"  OR "Equistasi device*"  OR "laser shoe*"  OR "laser cane*"  OR "smart glass*"  OR "head-mounted display*"  OR "HoloLens"  OR "Google Glass" ) |
| #3 | TS=( randomi*ed  OR randomly  OR randomization  OR randomisation OR "random allocation" OR "randomized controlled trial*"  OR "controlled clinical trial*" OR "clinical trial*"  OR "controlled trial*" OR placebo* OR "double blind*"  OR "single blind*") |
| #4 | #1 AND #2 AND #3 |

| **November 18, 2025** | **Cochrane Library (430)** |
| --- | --- |
| #1 | parkinson*  OR "Parkinson disease"  OR "Parkinson's disease"  OR "paralysis agitans"  OR (Lewy NEXT body NEXT parkinson*):ti,ab,kw |
| #2 | (wearable NEXT electronic NEXT device*)  OR (wearable NEXT device*)  OR (wearable NEXT technolog*)  OR (wearable NEXT sensor*)  OR (wearable NEXT system*)  OR (wearable NEXT cue*)  OR (body NEXT worn NEXT sensor*)  OR (body NEXT worn NEXT device*)  OR (exoskeleton*)  OR (robotic NEXT exoskeleton*)  OR (smartphone*)  OR (smart NEXT phone*)  OR (smartwatch*)  OR (smart NEXT watch*)  OR (mobile NEXT phone*)  OR (activity NEXT tracker*)  OR (fitness NEXT tracker*)  OR (acceleromet*)  OR (pedomet*)  OR (inertial NEXT sensor*)  OR (inertial NEXT measurement NEXT unit*)  OR (IMU)  OR (vibrotactile*)  OR (vibratory NEXT stimulat*)  OR (vibratory NEXT device*)  OR (textured NEXT insole*)  OR (shoe NEXT insole*)  OR (Equistasi)  OR (Equistasi NEXT device*)  OR (laser NEXT shoe*)  OR (laser NEXT cane*)  OR (smart NEXT glass*)  OR (head NEXT mounted NEXT display*)  OR (HoloLens)  OR (Google Glass):ti,ab,kw |
| #3 | (randomi*ed OR randomly OR randomization OR randomisation  OR placebo*  OR (randomi*ed NEXT controlled NEXT trial*)  OR (clinical NEXT trial*)  OR (controlled NEXT trial*)  OR (double NEXT blind*)  OR (single NEXT blind*)  OR crossover OR "cross-over"):ti,ab,kw |
| #4 | #1 AND #2 AND #3 |

| **November 18, 2025** | **Embase (1140)** |
| --- | --- |
| #1 | ('parkinson disease'/exp OR 'parkinsonian disorder'/exp  OR parkinson*:ti,ab,kw  OR parkinsonism:ti,ab,kw  OR parkinsonian*:ti,ab,kw  OR "paralysis agitans":ti,ab,kw  OR "Lewy body parkinson*":ti,ab,kw) |
| #2 | ('wearable electronic device'/exp  OR smartphone/exp OR 'cellular phone'/exp OR 'mobile phone'/exp  OR accelerometer/exp OR pedometer/exp OR exoskeleton/exp  OR 'virtual reality'/exp OR 'augmented reality'/exp  OR ((wearable OR 'body-worn' OR 'body worn') NEAR/3  (device* OR sensor* OR system* OR cue* OR technolog* OR equipment)):ti,ab,kw  OR (smartphone* OR 'smart phone*' OR smartwatch* OR 'smart watch*'  OR 'mobile phone*' OR cellphone* OR 'cell phone*'):ti,ab,kw  OR ('activity tracker*' OR 'fitness tracker*'):ti,ab,kw  OR (acceleromet* OR pedomet*):ti,ab,kw  OR ('inertial sensor*' OR 'inertial measurement unit*' OR IMU):ti,ab,kw  OR (vibrotactile* OR 'vibratory stimulation' OR 'vibratory stimulus' OR 'vibratory device*'):ti,ab,kw  OR ('textured insole*' OR 'shoe insole*'):ti,ab,kw  OR (Equistasi OR 'Equistasi device*'):ti,ab,kw  OR ('laser shoe*' OR 'laser cane*'):ti,ab,kw  OR ('smart glass*' OR 'smartglass*' OR 'head-mounted display*' OR HoloLens OR 'Google Glass'):ti,ab,kw) |
| #3 | ('randomized controlled trial'/exp  OR randomization/exp  OR 'double blind procedure'/exp  OR 'single blind procedure'/exp  OR 'crossover procedure'/exp  OR randomi*ed:ti,ab,kw  OR randomly:ti,ab,kw  OR randomization:ti,ab,kw  OR randomisation:ti,ab,kw  OR placebo*:ti,ab,kw  OR 'clinical trial*':ti,ab,kw  OR 'controlled trial*':ti,ab,kw  OR 'double blind*':ti,ab,kw  OR 'single blind*':ti,ab,kw  OR (crossover OR 'cross-over'):ti,ab,kw)  NOT ('animal'/exp NOT 'human'/exp) |
| #4 | #1 AND #2 AND #3 |

| **November 18, 2025** | **ClinicalTrials.gov (413)** |
| --- | --- |
| #1 | (parkinson OR "Parkinson Disease" OR "Parkinson's Disease" OR parkinsonian OR parkinsonism OR PD OR "paralysis agitans" OR "Lewy body parkinson*") |
| #2 | ("Wearable Electronic Devices" OR Smartphone OR "Cell Phone" OR "wearable electronic device*" OR "wearable device*" OR "wearable technolog*" OR "wearable sensor*" OR "wearable system*" OR "wearable cue*" OR "body-worn sensor*" OR "body-worn device*" OR "body worn sensor*" OR "body worn device*" OR exoskeleton* OR "robotic exoskeleton*" OR smartphone* OR "smart phone*" OR smartwatch* OR "smart watch*" OR "mobile phone*" OR "activity tracker*" OR "fitness tracker*" OR acceleromet* OR pedomet* OR "inertial sensor*" OR "inertial measurement unit*" OR IMU OR vibrotactile* OR "vibratory stimulation" OR "vibratory stimulus" OR "vibratory device*" OR "textured insole*" OR "shoe insole*" OR Equistasi OR "Equistasi device*" OR "laser shoe*" OR "laser cane*" OR "smart glass*" OR "head-mounted display*" OR HoloLens OR "Google Glass") |
| #3 | #1 AND #2 |
| #4 | Interventional [Study Type] AND Randomized [Study Design] |

**S2**

1. Model-based cueing-as-needed for walking in Parkinson’s disease: A randomized cross-over study
2. Effects of augmented reality cueing strategies on freezing of gait: The ELIMINATE FoG trial
3. Using a smartphone-based self-management platform to support medication adherence and clinical consultation in Parkinson’s disease
4. Comparative Effectiveness of mHealth-Supported Exercise Compared With Exercise Alone for People With Parkinson Disease: Randomized Controlled Pilot Study
5. A Smartphone Application as an Exploratory Endpoint in a Phase 3 Parkinson's Disease Clinical Trial: A Pilot Study
6. Effect of mobile health intervention for self-management on self-efficacy, motor and non-motor symptoms, self-management, and quality of life in people with Parkinson's disease: Randomized controlled trial
7. Stimulation of the Tibial nerve Repetitively to Improve Incontinence in Parkinson's Electronically (STRIPE trial): a randomised control trial of tibial nerve stimulation for bladder symptoms in Parkinson's disease using a self-contained wearable device
8. The Use of Telerehabilitation to Improve Movement-Related Outcomes and Quality of Life for Individuals With Parkinson Disease: Pilot Randomized Controlled Trial
9. Non-Immersive Virtual Reality Telerehabilitation System Improves Postural Balance in People with Chronic Neurological Diseases
10. Effects of a sequential square mat walking training program Fisior® on improving physical performance in older adults with Parkinson's disease: A pilot study
11. [On-Demand Cueing for Freezing of Gait in Parkinson's Disease: A Randomized Controlled Trial.](https://pubmed.ncbi.nlm.nih.gov/38486430/)
12. Comparing the efficacy of therapeutic Thai acupressure on plantar acupoints and laser cane therapy on freezing of gait in Parkinson's disease: a randomized non-inferiority trial
13. Evolution of the Motor Symptoms in Parkinson Disease under Auditory Stimulation
14. A wearable system for visual cueing gait rehabilitation in Parkinson's disease: a randomized non-inferiority trial
15. [Using gait robotics to improve symptoms of Parkinson's disease: an open-label, pilot randomized controlled trial.](https://pubmed.ncbi.nlm.nih.gov/35708047/)
16. Efficacy of Wearable Device Gait Training on Parkinson’s Disease: A Randomized Controlled Open-label Pilot Study

All reasons:

Not an RCT

Not a wearable-device intervention / no active wearable component

Wrong population (not PD / mixed not separable)

No eligible comparator

Outcomes not relevant / insufficient data for extraction

| **Section and Topic** | **Item #** | **Checklist item** | **Location where item is reported** |
| --- | --- | --- | --- |
| **TITLE** | | |  |
| Title | 1 | Identify the report as a systematic review. | Title page |
| **ABSTRACT** | | |  |
| Abstract | 2 | See the PRISMA 2020 for Abstracts checklist. | 1-2 |
| **INTRODUCTION** | | |  |
| Rationale | 3 | Describe the rationale for the review in the context of existing knowledge. | 2-3 |
| Objectives | 4 | Provide an explicit statement of the objective(s) or question(s) the review addresses. | 3 |
| **METHODS** | | |  |
| Eligibility criteria | 5 | Specify the inclusion and exclusion criteria for the review and how studies were grouped for the syntheses. | 5 |
| Information sources | 6 | Specify all databases, registers, websites, organisations, reference lists and other sources searched or consulted to identify studies. Specify the date when each source was last searched or consulted. | 4 |
| Search strategy | 7 | Present the full search strategies for all databases, registers and websites, including any filters and limits used. | 4-5 |
| Selection process | 8 | Specify the methods used to decide whether a study met the inclusion criteria of the review, including how many reviewers screened each record and each report retrieved, whether they worked independently, and if applicable, details of automation tools used in the process. | 6 |
| Data collection process | 9 | Specify the methods used to collect data from reports, including how many reviewers collected data from each report, whether they worked independently, any processes for obtaining or confirming data from study investigators, and if applicable, details of automation tools used in the process. | 6 |
| Data items | 10a | List and define all outcomes for which data were sought. Specify whether all results that were compatible with each outcome domain in each study were sought (e.g. for all measures, time points, analyses), and if not, the methods used to decide which results to collect. | 6-7 |
|  | 10b | List and define all other variables for which data were sought (e.g. participant and intervention characteristics, funding sources). Describe any assumptions made about any missing or unclear information. | 6-7 |
| Study risk of bias assessment | 11 | Specify the methods used to assess risk of bias in the included studies, including details of the tool(s) used, how many reviewers assessed each study and whether they worked independently, and if applicable, details of automation tools used in the process. | 7-8 |
| Effect measures | 12 | Specify for each outcome the effect measure(s) (e.g. risk ratio, mean difference) used in the synthesis or presentation of results. | 8 |
| Synthesis methods | 13a | Describe the processes used to decide which studies were eligible for each synthesis (e.g. tabulating the study intervention characteristics and comparing against the planned groups for each synthesis (item #5)). | 8 |
|  | 13b | Describe any methods required to prepare the data for presentation or synthesis, such as handling of missing summary statistics, or data conversions. | 8-9 |
|  | 13c | Describe any methods used to tabulate or visually display results of individual studies and syntheses. | 9 |
|  | 13d | Describe any methods used to synthesize results and provide a rationale for the choice(s). If meta-analysis was performed, describe the model(s), method(s) to identify the presence and extent of statistical heterogeneity, and software package(s) used. | 9 |
|  | 13e | Describe any methods used to explore possible causes of heterogeneity among study results (e.g. subgroup analysis, meta-regression). | 9 |
|  | 13f | Describe any sensitivity analyses conducted to assess robustness of the synthesized results. | 9-10 |
| Reporting bias assessment | 14 | Describe any methods used to assess risk of bias due to missing results in a synthesis (arising from reporting biases). | 10 |
| Certainty assessment | 15 | Describe any methods used to assess certainty (or confidence) in the body of evidence for an outcome. | 10 |
| **RESULTS** | | |  |
| Study selection | 16a | Describe the results of the search and selection process, from the number of records identified in the search to the number of studies included in the review, ideally using a flow diagram. | 10 |
|  | 16b | Cite studies that might appear to meet the inclusion criteria, but which were excluded, and explain why they were excluded. | Figure 1 |
| Study characteristics | 17 | Cite each included study and present its characteristics. | Table 1 |
| Risk of bias in studies | 18 | Present assessments of risk of bias for each included study. | Figure 2 |
| Results of individual studies | 19 | For all outcomes, present, for each study: (a) summary statistics for each group (where appropriate) and (b) an effect estimate and its precision (e.g. confidence/credible interval), ideally using structured tables or plots. | 11-13 |
| Results of syntheses | 20a | For each synthesis, briefly summarise the characteristics and risk of bias among contributing studies. | 11-13 |
|  | 20b | Present results of all statistical syntheses conducted. If meta-analysis was done, present for each the summary estimate and its precision (e.g. confidence/credible interval) and measures of statistical heterogeneity. If comparing groups, describe the direction of the effect. | 11-13 |
|  | 20c | Present results of all investigations of possible causes of heterogeneity among study results. | 11-13 |
|  | 20d | Present results of all sensitivity analyses conducted to assess the robustness of the synthesized results. | 11-13 |
| Reporting biases | 21 | Present assessments of risk of bias due to missing results (arising from reporting biases) for each synthesis assessed. | No have |
| Certainty of evidence | 22 | Present assessments of certainty (or confidence) in the body of evidence for each outcome assessed. | 14 |
| **DISCUSSION** | | |  |
| Discussion | 23a | Provide a general interpretation of the results in the context of other evidence. | 14 |
|  | 23b | Discuss any limitations of the evidence included in the review. | 16 |
|  | 23c | Discuss any limitations of the review processes used. | 16-17 |
|  | 23d | Discuss implications of the results for practice, policy, and future research. | 17 |
| **OTHER INFORMATION** | | |  |
| Registration and protocol | 24a | Provide registration information for the review, including register name and registration number, or state that the review was not registered. | CRD42024585686 |
|  | 24b | Indicate where the review protocol can be accessed, or state that a protocol was not prepared. | PROSPERO |
|  | 24c | Describe and explain any amendments to information provided at registration or in the protocol. | 19 |
| Support | 25 | Describe sources of financial or non-financial support for the review, and the role of the funders or sponsors in the review. | 19 |
| Competing interests | 26 | Declare any competing interests of review authors. | 19 |
| Availability of data, code and other materials | 27 | Report which of the following are publicly available and where they can be found: template data collection forms; data extracted from included studies; data used for all analyses; analytic code; any other materials used in the review. | The analytic code used for the meta-analyses and figure generation and the extracted data underlying the analyses are available from the corresponding author upon reasonable request. |

*From:*  Page MJ, McKenzie JE, Bossuyt PM, Boutron I, Hoffmann TC, Mulrow CD, et al. The PRISMA 2020 statement: an updated guideline for reporting systematic reviews. BMJ 2021;372:n71. doi: 10.1136/bmj.n71. This work is licensed under CC BY 4.0. To view a copy of this license, visit <https://creativecommons.org/licenses/by/4.0/>

**PRISMA-S Checklist**

| **Section/topic** | **#** | **Checklist item** | **Location(s) Reported** |
| --- | --- | --- | --- |
| **INFORMATION SOURCES AND METHODS** | | | |
| Database name | 1 | Name each individual database searched, stating the platform for each. | 4 (line114-118) |
| Multi-database searching | 2 | If databases were searched simultaneously on a single platform, state the name of the platform, listing all of the databases searched. | 4 (line119-120) |
| Study registries | 3 | List any study registries searched. | 4 (line116-118) |
| Online resources and browsing | 4 | Describe any online or print source purposefully searched or browsed (e.g., tables of contents, print conference proceedings, web sites), and how this was done. | 4 (line121-122) |
| Citation searching | 5 | Indicate whether cited references or citing references were examined, and describe any methods used for locating cited/citing references (e.g., browsing reference lists, using a citation index, setting up email alerts for references citing included studies). | 4 (line123-126) |
| Contacts | 6 | Indicate whether additional studies or data were sought by contacting authors, experts, manufacturers, or others. | 4 (line126-129) |
| Other methods | 7 | Describe any additional information sources or search methods used. | 4 (line128-129) |
| **SEARCH STRATEGIES** | | | |
| Full search strategies | 8 | Include the search strategies for each database and information source, copied and pasted exactly as run. | 5 (line142-144) |
| Limits and restrictions | 9 | Specify that no limits were used, or describe any limits or restrictions applied to a search (e.g., date or time period, language, study design) and provide justification for their use. | 5 (line137-140) |
| Search filters | 10 | Indicate whether published search filters were used (as originally designed or modified), and if so, cite the filter(s) used. | 4 (line133) |
| Prior work | 11 | Indicate when search strategies from other literature reviews were adapted or reused for a substantive part or all of the search, citing the previous review(s). | 4 (line134-136) |
| Updates | 12 | Report the methods used to update the search(es) (e.g., rerunning searches, email alerts). | 4 (line120-122) |
| Dates of searches | 13 | For each search strategy, provide the date when the last search occurred. | 4 (line116-117) |
| **PEER REVIEW** | | | |
| Peer review | 14 | Describe any search peer review process. | 5 (line144-145) |
| **MANAGING RECORDS** | | | |
| Total Records | 15 | Document the total number of records identified from each database and other information sources. | Figure 1 |
| Deduplication | 16 | Describe the processes and any software used to deduplicate records from multiple database searches and other information sources. | Figure 1 |
|  |  |  |  |
| PRISMA-S: An Extension to the PRISMA Statement for Reporting Literature Searches in Systematic Reviews | | |  |
| Rethlefsen ML, Kirtley S, Waffenschmidt S, Ayala AP, Moher D, Page MJ, Koffel JB, PRISMA-S Group. | | |  |
| Last updated February 27, 2020. | |  |  |
